# Hyperphosphatemia as an independent risk factor for coronary artery calcification progression in peritoneal dialysis patients

**DOI:** 10.1186/s12882-015-0103-8

**Published:** 2015-07-18

**Authors:** Da Shang, Qionghong Xie, Xiaolin Ge, Huanqing Yan, Jing Tian, Dingwei Kuang, Chuan-Ming Hao, Tongying Zhu

**Affiliations:** Division of Nephrology, Huashan Hospital, Fudan University, 12 Wulumuqi Road (middle), Shanghai, 200040 China; Division of Nephrology, Huashan Hospital Baoshan Branch, Fudan University, Shanghai, 200443 China

**Keywords:** Coronary artery calcification, Peritoneal dialysis, Hyperphosphatemia, ESRD

## Abstract

**Background:**

Coronary artery calcification (CAC) is associated with cardiovascular mortality in end-stage renal disease (ESRD) patients. The present study aimed to identify modifiable risk factors for CAC progression in peritoneal dialysis (PD) patients.

**Methods:**

Adult patients who received regular PD for more than 6 months and underwent a series of coronary artery calcification score (CaCS) measurements by multislice spiral computed tomography (MSCT) with an interval of ≥ 6 months were included in this observational cohort study. The demographic characteristics and clinical data, including laboratory data and adequacy of PD, were collected. Curve estimation was used to fit the straight line and obtain the slope. Binary logistic regression was performed to identify the independent risk factors for CAC progression in the PD patients, and multivariate linear regression was conducted to identify factors associated with hyperphosphatemia.

**Results:**

A total of 207 adult patients on PD (116 men, 56.0 %) with a mean age of 59.8 ± 15.9 years were recruited to this study, and 157 of them (75.8 %) received three or more CaCS assessments. The patients were divided into a slow group (*n* = 137) and a rapid group (*n* = 70) according to the linear regression slope or the average speed of development. The follow-up time was 33.0 ± 18.8 months. Multivariate logistic regression revealed that age and serum phosphate level were independent risk factors for CAC progression after adjustments. Multivariate linear regression revealed that hyperphosphatemia was associated with elevations in the transferrin and serum albumin levels and normalized protein catabolic rate (nPCR) and reductions in the hemoglobin level, residual Ccr, and PD Ccr.

**Conclusions:**

Hyperphosphatemia is an independent risk factor for CAC progression, and the serum phosphate level may be associated with protein intake and PD adequacy. These results provide important information for the clinical management of ESRD patients.

## Background

Cardiovascular disease (CVD) is the leading cause of death in patients on peritoneal dialysis (PD), and it is more frequent in these patients than in the general population. In addition, cardiovascular mortality is up to 100-fold higher in these patients than in the general age-matched population, especially in younger patients [1, 2]. Although traditional risk factors for CVD in the general population, including hypertension, diabetes mellitus and hyperlipidemia, are also important for PD patients, other factors specific to end-stage renal disease (ESRD) and/or PD also play important roles [3, 4]. The presence, extent and progression of vascular calcification are strongly associated with CVD and all-cause mortality in ESRD patients [5–8]. Assessment of the coronary artery calcification score (CaCS) by computerized tomography [9], a noninvasive imaging technique, has been suggested for evaluation of vascular calcification and has been demonstrated to have a notable predictive value for CVD.

Although coronary artery calcification (CAC) is an independent prognostic predictor for ESRD patients [5–8], the risk factors associated with CAC progression remain uncertain. In the general population, traditional factors, such as diabetes, hypertension and dyslipidemia, have been proposed to contribute to its progression. In contrast with the general population, ESRD patients are characterized by complex complications, such as hypertension, mineral metabolism disorder, micro-inflammation and a poor nutritional state. Age, hypercalcemia, hyperphosphatemia, PD duration, hyperlipidemia and inflammation are associated with CAC progression [8, 10–14].

Hyperphosphatemia is common in ESRD patients on PD. Together with dysregulated calcium, parathyroid hormone and vitamin D levels, hyperphosphatemia contributes to mineral and bone diseases [15–17]. Epidemiological studies have suggested that hyperphosphatemia is associated with increased cardiovascular events and all-cause mortality and that a decrease in the serum phosphate level is linked to improved survival [18–21]. Upon exposure to high phosphate levels, bone-like cells form in arteries, resulting in extensive calcification [22, 23]. Hyperphosphatemia has been associated with increases in vessel wall thickness and arterial stiffness [24]. However, the association between hyperphosphatemia and CAC progression remains controversial in ESRD patients on PD [10, 11, 13, 14, 25].

The aim of this study was to examine the potential risk factors for CAC progression in ESRD patients on PD. In addition, we sought to determine whether hyperphosphatemia is an independent risk factor for CAC progression in these patients and to analyze the factors associated with hyperphosphatemia.

## Methods

### Study population

Adult patients on PD treated at Huashan Hospital Fudan University in China from January 2007 to October 2012 were included in this observational cohort study. Patients who received regular PD for more than 6 months and underwent CaCS measurements at least twice with an interval of ≥ 6 months were analyzed. Patients were excluded if they had acute infection, unstable CVD or were clinically unstable, with a life expectancy of shorter than 6 months. Patients whose active disease was stable for over 2 months were eligible for this study. This study was approved by the ethics committee of Huashan Hospital at Fudan University. All of the patients gave written informed consent.

### Coronary artery calcification score

CAC was assessed by multislice spiral computed tomography (MSCT) and recorded as the Agatston score (CaCS). In this method, the heart was scanned over a period of 20 to 30 s, with a distance of 3 mm between each slice. The software built into this equipment detected calcified lesions with a density of at least 130 Hounsfield units and a minimal area of 0.5 mm^2^. The acquisition time for this method was 125 milliseconds. For imaging reconstruction, this method used data obtained during the diastolic phase of the cardiac cycle. The total radiation dose throughout the entire procedure was 1.25 millisieverts (mSV). Calculations of the total CaCS were based on formulas that included measurements of the total volume, area and mean and maximum densities of the calcified lesions. Individual CaCS were calculated for the left main coronary artery, descending branch of the left coronary artery, circumflex branch of the left coronary artery and right coronary artery. Then, the scores were summed to calculate the total coronary CaCS. The final score is expressed in modified Agatston units.

### Evaluation of coronary artery calcification progression

All patients with a CaCS of 0 during follow-up were placed into the slow progression group. For the other patients, the dependent variable, Y_n_ = the square root of CaCS_n_ (SQRCaCS_n_), and the independent variable, T_n_ = D_n_ - D_1_ (T_n_ represents the time interval between the n^th^ and 1^st^ CaCS assessment), were calculated. If Y_n_ = f(T_n_) fitted the linear relationship for each patient, then V = f(T_n_)/T_n_ was calculated. If not, the average speed of development represented by the geometric average, $\sqrt[t]{Ca{C}_n/CaC{S}_1},$ was calculated. The patients with a V ≥ 0.3 (median) or a geometric average ≥ 1.03 (median) were placed into the rapid CAC progression group, and those with a V < 0.3 or a geometric average < 1.03 were classified as the slow group.

### Laboratory data

Demographic characteristics (age, gender, height, weight and smoking status) and comorbidities (diabetes mellitus, hypertension and CVD) were recorded at baseline. Laboratory measurements, including calcium-phosphate metabolism (the serum phosphate level, adjusted calcium level, as calculated by [measured calcium + (4 – albumin g/dL) × 0.8], and intact parathyroid hormone [iPTH] level), lipid (cholesterol, triglycerides, low-density lipoprotein, high-density lipoprotein [HDL] and lipoprotein a), inflammation marker (high-sensitivity C-reactive protein and fibrinogen), hemoglobin, albumin, transferrin, serum iron and pro-brain natriuretic peptide (pro-BNP) levels, were collected every 3 months. The average values of these indexes were calculated between CaCS measurements. PD adequacy was evaluated every 6 months using Baxter PD Adequest 2.0 software (Baxter Healthcare Corporation, Deerfield, IL, USA), and the averages of these parameters, including the total Kt/V, residual Kt/V, total clearance of creatinine (Ccr), residual Ccr and normalized protein catabolic rate (nPCR), were obtained. In addition, the clearance of phosphate (Cp) and calcium (Cca) by the dialysate, adjusted for the body surface area (BSA), the peritoneal permeability for phosphate (D4/P phosphate = the phosphate concentration in the dialysate at the 4^th^ hour / the plasma phosphate concentration) and the D/P phosphate or calcium (the mean phosphate or calcium concentration in the dialysate / the plasma phosphate or calcium concentration), were also obtained.

### Statistical analysis

Statistical analysis of the data was performed using SPSS software, version 17.0. Continuous variables are expressed as the mean ± SD or as the median and quartile range, and categorical variables are expressed as percentages. Groups were compared using the independent samples *t*-test or Mann–Whitney *U* test for continuous variables and Pearson’s chi-square test or Fisher’s exact test for categorical variables. Curve estimation was performed for linear regression analysis. Binary logistic regression was used to identify the independent risk factors for CAC in the PD patients. Covariates that were considered risk factors for the endpoints in univariate analysis were used for adjustments. The odds ratios (ORs) for the endpoints are expressed per 10-year age interval, 100-unit increase in the baseline CaCS, and 0.1 g/dL increase in the albumin level. Univariate linear regression was performed to identify the factors potentially related to hyperphosphatemia. Multivariate linear regression was conducted to verify the risk factors and covariates associated with hyperphosphatemia identified in univariate analysis and for adjustments. The associations between hyperphosphatemia and other factors were expressed by unstandardized and standardized coefficients. All of the statistical tests were 2-sided, and the differences were considered statistically significant at a P-value of < 0.05.

## Results

### Patients’ characteristics

**Fig. 1:**
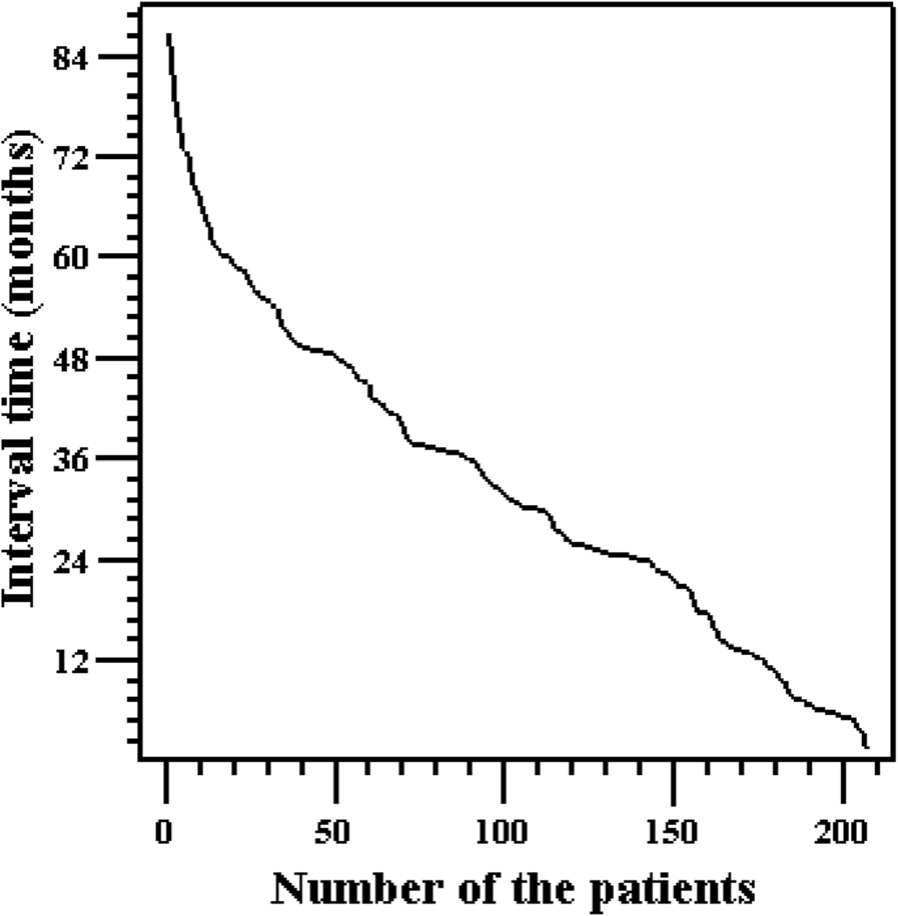
The relationship between the interval and number of patients

A total of 207 adult patients on PD (116 men, 56.0 %) with a mean age of 59.8 ± 15.9 years were recruited for this study. The follow-up time was 33.0 ± 18.8 months. A total of 70 patients started PD before initiation of the study (from 0.5 - 9 years prior). The average duration of PD therapy for all of the patients at baseline was 1.08 (−0.26 - 7.10) months, and there were no significant differences between the groups. Sixty-eight patients were followed for less than 2 years, 89 for more than 2 years but less than 4 years, and 50 for more than 4 years (Fig. 1). One hundred and fifty-seven (75.8 %) patients received ≥ 3 CaCS assessments, 116 (56 %) received ≥ 4 and 69 (33.3 %) had ≥ 5. In addition, 137 (66.2 %) patients received baseline CaCS examination from 1 month before PD to 6 months after PD. The basic CaCS was 0 for 84 (40.6 %) patients, 1 to 100 for 53 (25.6 %) patients, and ≥ 100 for 70 (33.8 %) patients.

More than 90 % of the patients in our study received continuous ambulatory peritoneal dialysis (CAPD), while others received daytime ambulatory peritoneal dialysis (DAPD). The peritoneal dialysates used for all patients were Dianeal PD4 (1.25 mmol/L calcium, 2 L) and Dianeal PD2 (1.75 mmol/L calcium, 2 L) from Baxter Healthcare Corporation. Approximately 93 % of the patients received 3 changes (62 %) or 4 changes (31 %) of dialysate, and the long and short dwelling times were 12 h and 4 to 6 h, respectively. No icodextrin was used at our center because it is not available in China. There were no significant differences in the use of CAPD or Dianeal PD4/PD2 between the rapid and slow groups.

Eight patients had a history of cardiovascular events (CVEs) at baseline, and there were no significant differences between the groups (1/70 vs. 7/137, *p* = 0.195). During the follow-up period, 57 patients died and 54 experienced CVEs. Among the patients who died, 25 died of a CVE and 14 died of infection. A total of 25 patients died in the rapid group (70 patients), and 32 died in the slow group (137 patients).

Among the patients, 61 (29.5 %) had a history of diabetes at the beginning of the study, and 78 (37.7 %) had a history of smoking. Thirty-nine (18.8 %) patients had a serum phosphate level of > 5.5 mg/dL, and 117 (56.5 %) had a level of > 4.5 mg/dL. Among these patients, 133 (64.3 %) received calcium carbonate treatment (78/137 vs. 55/70; *p* = 0.002), and 143 (69.1 %) received calcitriol treatment. Forty-three (20.8 %) patients had a serum cholesterol level of >200 mg/dL, and 138 (66.7 %) received lipid-lowering drugs. All of the treatments were strictly administered according to the K/DOQI or KDIGO guidelines and were not influenced by this study. The mean hemoglobin level was 103.6 ± 13.1 g/L, and the mean nPCR was 0.87 ± 0.16. The median serum albumin level was 3.47 g/dL. In total, 116 (56.0 %) patients had an average residual renal Ccr of > 20 L/week, 97 (46.9 %) had an average total Ccr of > 65 L/week, and 180 (87.0 %) had a total Ccr of > 50 L/week. The mean D4/P phosphate was 0.53 ± 0.12, which was strongly associated with the D4/P creatinine, and the D/P phosphate was 0.59 ± 0.16.

### Hyperphosphatemia is an independent risk factor for CaCS progression

Sixty-three patients with a CaCS of 0 were placed into the slow group, with a mean follow-up time of 32.7 ± 19.1 months, which was similar to that of the other 144 patients (33.1 ± 18.7 months; *p* = 0.898). Among the 144 patients, the progression of calcification fitted the linear relationship in 63, and it did not in 81. The 31 patients with a V < 0.3 and the 43 patients with a geometric average < 1.03 were also placed into the slow group. The remaining patients (*n* = 70) were classified as the rapid group. Univariate analysis revealed that the patients with rapid progression were older, had a higher baseline CaCS and had a higher absolute change in the calcification score at the end of the observation period versus the baseline (absolute CaCS) compared with the slow group. Further, the patients with rapid progression had a higher body mass index (BMI) and serum phosphate, serum creatinine and lipoprotein a levels but lower HDL and hemoglobin levels. With regard to inflammation, the patients with rapid progression had a higher hs-CRP level. With regard to PD adequacy, the patients with slow progression had elevations in total Ccr and Kt/V, residual renal function and D/P phosphate. Unexpectedly, the patients with slow progression had a higher D/P phosphate, but no significant differences were found in the excretion of calcium and phosphate in the dialysate between the slow and rapid groups. No significant correlation in gender, the cause of ESRD, the iPTH, cholesterol, or triglyceride level, nPCR, PD-Ccr or Kt/V was observed between the slow and rapid progression groups (Table 1). Multivariate analysis revealed that age (*p* = 0.023) and serum phosphate level (*p* = 0.031) were independent risk factors for CAC progression after adjusting for gender, basic CaCS, lipoprotein a, HDL, hemoglobin, serum creatinine, and hs-CRP levels, total Ccr, residual Ccr and D/P phosphate (Table 2).

**Table 1:** Clinical characteristics of the peritoneal dialysis patients

	Total (*n* = 207)	Slow Group (*n* = 137)	Rapid Group (*n* = 70)	*P*-value
Follow-up time (months)	33.0 ± 18.8	33.9 ± 19.7	31.2 ± 16.7	0.299
Age (years)	59.8 ± 15.9	58.0 ± 17.0	63.4 ± 13.1	0.012
BMI (kg/m^2^)	23.56 ± 3.72	22.87 ± 3.69	24.90 ± 3.40	<0.001
Gender (male)	56.0 %	52.60 %	62.90 %	0.184
Cause of ESRD				0.311
Glomerulonephritis	59.9 %	61.3 %	57.1 %	
Diabetes mellitus	29.5 %	24.8 %	38.6 %	
Polycystic kidney	2.4 %	2.9 %	1.4 %	
Hypertension	2.4 %	3.6 %	0 %	
Others	5.8 %	7.3 %	2.9 %	
Smoking	37.7 %	34.30 %	44.30 %	0.175
Diabetes mellitus	29.5 %	24.80 %	38.60 %	0.053
CVD before PD	3.9 %	5.1 %	1.4 %	0.195
PD duration before CAC (months)	1.08 (−0.26-7.10)	1.08 (−0.31-6.39)	1.18 (−0.16-10.87)	0.380
Baseline CaCS	13.8 (0–239.0)	0 (0–175.2)	71.4 (1.5-304.0)	0.001
Absolute CaCS	61.7 (0–370.6)	0 (0–115.5)	381.8 (103.0-907.4)	<0.001
nPCR (g/kg*d)	0.87 ± 0.16	0.88 ± 0.16	0.84 ± 0.14	0.107
Total protein (g/dL)	6.55 ± 0.53	6.56 ± 0.51	6.55 ± 0.58	0.931
Albumin (g/dL)	3.47 (3.25-3.70)	3.50 (3.28-3.75)	3.42 (3.22-3.59)	0.052
Cholesterol (mg/dL)	173 (157–197)	172 (154–197)	173 (159–197)	0.918
Triglycerides (mg/dL)	155 (114–209)	151 (113–210)	163 (122–209)	0.386
LDL (mg/dL)	95.0 ± 23.2	94.0 ± 23.7	96.8 ± 22.4	0.424
Lipoprotein a (mg/dL)	198 (125–324)	182 (110–319)	258 (168–325)	0.01
HDL (mg/dL)	37.1 (32.1-43.3)	38.3 (32.7-46.4)	36.5 (32.0-40.3)	0.03
Serum creatinine (mg/dL)	9.09 ± 3.26	8.68 ± 3.22	9.90 ± 3.22	0.011
Pro-BNP (pg/mL)	5094 ± 6592	4542 ± 5625	6188 ± 8121	0.106
Adjusted total Ccr (L/W)	63.6 (54.3-76.1)	56.6 (67.0-83.1)	58.0 (51.1-67.2)	<0.001
Adjusted PD Ccr (L/W)	40.0 ± 8.9	39.9 ± 8.9	40.2 ± 9.0	0.785
Adjusted residual Ccr (L/W)	29.0 ± 25.3	32.9 ± 27.1	21.2 ± 19.1	<0.001
Total Kt/V	2.03 ± 0.40	2.11 ± 0.42	1.89 ± 0.32	<0.001
PD Kt/V	1.48 ± 0.37	1.47 ± 0.38	1.49 ± 0.35	0.772
Residual Kt/V	0.45 (0.16-0.79)	0.55 (0.22-0.86)	0.32 (0.13-0.56)	0.002
D/P phosphate	0.59 ± 0.16	0.61 ± 0.18	0.55 ± 0.11	0.007
D/P calcium	0.58 (0.50-0.62)	0.58 (0.50-0.62)	0.58 (0.48-0.62)	0.58
Cp (mmol/d/1.732)	4.58 ± 1.77	4.39 ± 1.78	4.93 ± 1.71	0.069
Cca (mmol/d/1.732)	0.64 ± 0.61	0.59 ± 0.58	0.74 ± 0.66	0.132
D4/P phosphate	0.53 ± 0.12	0.54 ± 0.12	0.51 ± 0.13	0.161
iPTH (ng/dL)	297 (195–416)	286 (186–420)	323 (212–412)	0.329
Phosphorus (mg/dL)	4.72 ± 0.97	4.59 ± 0.98	4.99 ± 0.88	0.005
Adjusted calcium (mg/dL)	9.13 ± 0.67	9.15 ± 0.60	9.09 ± 0.79	0.51
Hemoglobin (g/L)	103.6 ± 13.1	105.1 ± 13.5	100.5 ± 11.8	0.015
hs-CRP (mg/dL)	0.19 (0.09-0.34)	0.15 (0.07-0.32)	0.24 (0.17-0.41)	0.001
CAPD	91.2 %	93.5 %	88.3 %	0.092
Calcium carbonate use	64.3 %	56.90 %	78.60 %	0.002
Calcitriol use	69.1 %	67.90 %	71.40 %	0.637
Lipid-lowering drug use	66.7 %	62.80 %	74.30 %	0.119

**Table 2:** Multivariate analyses of the selected possible risk factors for CaCS progression in PD patients

	OR	P
Age (per 10 years)	1.503 (1.059-2.133)	0.023
Gender	2.021 (0.778-5.245)	0.148
BMI (kg/m^2^)	1.134 (0.988-1.302)	0.073
Baseline CaCS (per 100)	1.004 (0.955-1.056)	0.873
Total Ccr (L/W)	1.005 (0.935-1.080)	0.898
Residual Ccr (L/W)	0.983 (0.930-1.039)	0.539
D/P phosphate	0.142 (0.007-3.051)	0.213
Phosphorus (mg/dL)	2.043 (1.068-3.910)	0.031
Hemoglobin (g/dL)	0.983 (0.950-1.018)	0.337
Lipoprotein a (mg/dL)	1.001 (1.000-1.003)	0.138
HDL (mg/dL)	0.986 (0.939-1.035)	0.569
hs-CRP (mg/dL)	1.663 (0.377-7.338)	0.502
Serum creatinine (mg/dL)	0.980 (0.784-1.224)	0.856

In addition, we analyzed the serum phosphate levels in the 63 patients with a CaCS of 0 throughout the study, and compared them with those of the slow progressors (those with a CaCS of 0 throughout the study were excluded) and rapid progressors [26]. In univariate analysis, the serum phosphate level in the rapid progressors (5.0 ± 0.88 mg/dL) was significantly higher than that of the slow progressors (4.37 ± 0.86 mg/dL, *p* < 0.001), consistent with our previous results. However, the serum phosphate level in the patients who did not experience CAC during study (4.84 ± 1.06 mg/dL) did not differ compared with that in the rapid progressors (*p* = 0.36) and was significantly higher than that in the slow progressors. Multivariate analysis revealed that serum phosphate level was an independent risk factor for CAC progression after adjusting for age, gender, basic CaCS, total Ccr, hemoglobin level, and BMI (*p* = 0.048). These findings suggest that the serum phosphate level is not a risk factor contributing to the initiation of vascular calcification.

### The risk factors for hyperphosphatemia

Linear regression was performed to analyze associations between the average serum phosphate level and other factors. Univariate analysis showed that nutritional markers (serum transferrin, BUN, and albumin levels and nPCR), calcium phosphate metabolism (the iPTH level, Cp, Cca and D/P phosphate) and the pro-BNP level were positively associated with the serum phosphate level. In contrast, age, the hemoglobin level and PD adequacy (total Ccr and Kt/V, residual Ccr and Kt/V) were inversely associated with this level, and the dialysis Ccr and Kt/V, BMI, and adjusted calcium, hs-CRP and lipid levels were not significantly associated with it (Table 3). Multivariate analysis revealed that the patients with hyperphosphatemia were younger (*p* = 0.003). Other factors found to be independently associated with hyperphosphatemia were elevations in the transferrin (*p* = 0.007) and serum albumin levels (*p* = 0.049) and nPCR (*p* < 0.001) and decreases in the hemoglobin level (*p* = 0.015), residual Ccr (*p* < 0.001), and PD Ccr (*p* < 0.001) (Table 4).

**Table 3:** Univariate analysis for the selected possible predictors of phosphate in PD patients

	Unstandardized Coefficients	Standardized Coefficients	*P*-value
Age (per 10 years)	−0.247 ± 0.039	−0.407	<0.001
Gender	0.157 ± 0.135	0.081	0.247
Baseline CaCS (per 100 ng/L)	−0.008 ± 0.007	−0.086	0.22
BMI (kg/m^2^)	0.019 ± 0.018	0.072	0.304
Transferrin (g/L)	0.123 ± 0.043	0.195	0.005
BUN (mmol/L)	0.015 ± 0.005	0.231	0.001
Albumin (g/dL)	0.461 ± 0.117	0.265	<0.001
nPCR (g/kg · d)	1.710 ± 0.418	0.275	<0.001
Hemoglobin (g/L)	−0.027 ± 0.005	−0.359	<0.001
CO2CP (mmol/L)	−0.090 ± 0.025	−0.245	<0.001
Adjusted calcium (mg/dL)	−0.138 ± 0.101	−0.095	0.173
iPTH (per 100 ng/L)	0.122 ± 0.031	0.272	<0.001
hs-CRP (mg/dL)	−0.001 ± 0.003	−0.019	0.793
Pro-BNP (ng/mL)	0.033 ± 0.010	0.245	0.001
Cholesterol (per 100 mg/dL)	0.097 ± 0.076	0.089	0.2
Triglycerides (per 100 mg/dL)	−0.022 ± 0.071	−0.022	0.755
LDL (per 100 mg/dL)	−0.147 ± 0.290	−0.035	0.614
HDL (per 100 mg/dL)	−0.442 ± 0.289	−0.106	0.128
Lipoprotein a (per 100 mg/dL)	0.006 ± 0.003	0.015	0.838
Total Ccr (L/W)	−0.017 ± 0.003	−0.372	<0.001
PD Ccr (L/W)	−0.013 ± 0.008	−0.121	0.082
Residual Ccr (L/W)	−0.010 ± 0.003	−0.266	<0.001
Total K/tv	−0.985 ± 0.152	−0.412	<0.001
PD K/tv	−0.317 ± 0.181	−0.122	0.081
Residual K/tv	−0.465 ± 0.131	−0.241	<0.001
Cp (mmol/d/1.73^2^)	0.297 ± 0.033	0.581	<0.001
Cca (mmol/d/1.73^2^)	0.313 ± 0.117	0.21	0.008
D4/P phosphate	−1.265 ± 0.586	−0.174	0.033
D/P phosphate	0.083 ± 0.462	0.015	0.858
D/P calcium	−0.105 ± 0.099	−0.086	0.292

**Table 4:** Multivariate analysis of selected factors associated with hyperphosphatemia in PD patients

	UnstandardizedCoefficients	Standardized Coefficients	*P*-value
Age (per 10 years)	−0.122 ± 0.040	−0.211	0.003
Gender	0.051 ± 0.121	0.028	0.671
Transferrin (g/L)	0.098 ± 0.036	0.162	0.007
Albumin (g/dL)	0.188 ± 0.095	0.128	0.049
nPCR (g/kg/d)	1.560 ± 0.416	0.265	<0.001
Hemoglobin (g/L)	−0.011 ± 0.005	−0.165	0.015
iPTH (per 100 ng/L)	0.049 ± 0.026	0.120	0.061
Residual Ccr (L/W)	−0.021 ± 0.003	−0.590	<0.001
PD Ccr (L/W)	−0.037 ± 0.009	−0.355	<0.001
D4/P phosphate	−0.858 ± 0.462	−0.119	0.065

## Discussion

The aim of this study was to determine the risk factors for and whether hyperphosphatemia was independently associated with CAC progression. We found that age and hyperphosphatemia were independent risk factors for CAC progression after adjusting for basic CaCS, lipid levels, inflammation, D/P phosphate and PD adequacy. The serum phosphate level was positively associated with nutritional markers, including the transferrin and serum albumin levels and nPCR, but negatively associated with age, the hemoglobin level and PD adequacy, including the peritoneal and residual renal Ccr. These results suggest that hyperphosphatemia accelerates the progression of vascular calcification, and treatments that lower the serum phosphate level, including restriction of phosphate intake and protection of residual renal function, may be beneficial for ESRD patients receiving PD.

In addition, our study showed that diabetes mellitus had a trend of a higher prevalence (*p* = 0.053) in the rapid progression group, as determined by univariate analysis. This finding may be associated with the stimulation of glycoxidation and hypoxia during arterial hyperglycemia. Further studies are required to identify the metabolic factors that are related to diabetes and contribute to aortic calcification in diabetic PD patients [27]. Rroji M’s study has shown that residual renal function in PD patients contributes significantly to the maintenance of phosphate balance and may explain the lower prevalence of valve calcification in PD patients compared with HD patients. Residual renal function not only allows for small solute clearance but also plays important roles in maintaining the fluid balance and phosphate control and removing middle molecule uremic toxins [28, 29]. In our study, dialysis adequacy was associated with CAC progression, as shown by univariate analysis, highlighting the importance of RRF in the control of vascular calcification.

Vascular calcification is significantly associated with the prognosis of ESRD patients [5–8]; therefore, some studies have focused on the risk factors for vascular calcification progression. In Noordzij’s study [14], 237 patients on PD/HD were enrolled, and chest X-ray was performed to evaluate the progression of aortic calcification during follow-up (mean of 2.3 years). They found that aortic calcification progressed in almost one-third of the patients. In addition, age, hypercalcemia and hyperparathyroidism were associated with an increased risk of progression, while hyperphosphatemia was not associated with progression. Another study on aortic calcification included 184 PD/HD patients and found that dialysis duration and the basic calcification score were associated with progression [11]. Studies on CAC have reported that the levels of cholesterol, LDL and inflammation markers, such as CRP, are positively associated, but that serum albumin is inversely associated, with progression [8, 12, 13, 30]. A few studies have shown that BMI and hyperphosphatemia are independent risk factors for CAC progression [25]. Hence, the risk factors for vascular calcification remain unclear. In our study, age, basic CaCS, BMI, phosphate, HDL, lipoprotein a, hemoglobin, and hs-CRP levels, D/P phosphate and dialysis adequacy were associated with CAC progression, as shown by univariate analysis. However, the results of multivariate analysis indicated that only age and serum phosphate level were independent risk factors.

Chronic hyperphosphatemia in patients undergoing dialysis is associated with elevations in cardiovascular morbidity and mortality [18–21]. Since the late 1980s, the focus of nephrologists has drastically shifted from bone damage and extravascular calcification of soft tissues to cardiovascular damage related to hyperphosphatemia. The mechanisms of this damage are thought to be linked to the ability of phosphate to enhance vascular calcification [31]. Extracellular phosphate promotes the mineralization of vascular smooth muscle cells in both dosage- and time-dependent manners by inducing osteoblastic differentiation factors [32]. Whether hyperphosphatemia is associated with the progression of vascular calcification remains controversial in clinical studies. Noordzij’s study [14] has revealed that hypercalcemia and hyperparathyroidism are associated with an increased risk of progression but that hyperphosphatemia is not. Stompor’s study [25] has shown that hyperphosphatemia is associated with CAC progression by univariate analysis but that it is not an independent risk factor. Other studies have also shown that hyperphosphatemia is not a risk factor for CAC progression [8, 10–12, 30]. In contrast with the above studies, we found that hyperphosphatemia was an independent risk factor for CAC progression after adjusting for age, gender, BMI, baseline CaCS, HDL, lipoprotein a, hemoglobin, serum creatinine, and hs-CRP levels, total Ccr, residual Ccr and D/P phosphate. This disparity among results may be attributed to the following factors: 1. the serum phosphate level was an average level measured during the follow-up period in our study, and thus it better represented the actual level compared with the use of only the baseline level. 2. The sample size of our study was larger and the follow-up time was longer than those in the previous studies. 3. The patients recruited to our study received a series of CaCS assessments, which was superior to the evaluation of calcification by two CaCS measurements. 4. The heterogeneity of our study population was relatively small because all of the patients were stable PD patients, and approximately two-thirds of them were recruited to our cohort at the beginning of PD treatment.

Our univariate analysis showed that calcium carbonate use was associated with CAC progression (78/137 vs. 55/70; *p* = 0.002). Because only patients with hyperphosphatemia were prescribed calcium carbonate in our cohort, we considered them as two dependent variables. Therefore we did not include calcium carbonate in multivariate analysis. However, we analyzed the correlation of the serum phosphate level and CaCS progression by partial correlation analysis, adjusting for the calcium carbonate level, age, BMI, sex, renal residual Ccr and basic CaCS. We found that the serum phosphate level was associated with CaCS progression. Therefore, our results suggest the serum phosphate level is an independent risk factor for CaCS progression, but they do not exclude the possible association of the calcium carbonate level with the CaCS.

If hyperphosphatemia is independently associated with the progression of vascular calcification, then control of the serum phosphate level may be beneficial for improving the prognosis of ESRD patients. Therefore, studies on the risk factors for hyperphosphatemia are useful for achieving serum phosphate control. In a multi-center observational study of phosphate control in PD patients [33], the serum phosphate level was found to be positively correlated with the serum albumin and iPTH levels and negatively correlated with age. Another study [28] showed that hyperphosphatemia was positively correlated with BMI, the iPTH level and nPNA (normalized protein equivalent of nitrogen appearance) and inversely correlated with PD Ccr/Kt/v and residual GFR. Similar to these studies, we found that hyperphosphatemia was positively associated with nPCR and the albumin and transferrin levels and negatively associated with age and PD adequacy. These findings may suggest that the serum phosphate level is mainly determined by dietary phosphate intake and PD adequacy. Therefore, the restriction of phosphate intake and increase in PD adequacy may be useful for slowing the progression of vascular calcification. In addition, the level of serum phosphate was negatively associated with that of hemoglobin, a finding that may be attributed to the increased levels of polyamines caused by hyperphosphatemia, which can inhibit erythropoiesis [34, 35].

There are some limitations to our study. First, this report was an observational, single-center and relatively small study. Our results only describe hyperphosphatemia as an independent risk factor for CAC progression, and we only analyzed factors related to the serum phosphate level. We did not examine whether the control of serum phosphate could slow CAC progression. Second, the follow-up times for the patients in this study varied from 6 to 87 months, and approximately 15 % of them were followed up for less than 12 months. CAC progression was not linear in a portion of the patients; thus, we calculated the velocity according to the average speed of development represented by the geometric average, which may have introduced error.

## Conclusions

In conclusion, our results indicate that hyperphosphatemia is an independent risk factor for CAC progression, that high BMI has a trend of increased prevalence in PD patients and that the serum phosphate level is positively associated with nutritional markers and PD adequacy, indicating that a high daily phosphate intake and PD inadequacy may accelerate CAC progression. Control of serum phosphate might slow the progression of vascular calcification and improve the prognosis of PD patients. However, these results require further examination by interventional trials.

### Strengths

First, the lab data comprised the average levels measured during the follow-up period, which better represented the actual levels. Second, the sample size of our study was larger, and the follow-up time was longer. Third, the patients recruited for our study received a series of CaCS assessments, which is superior to the evaluation of calcification by two CaCS measurements.

### Limitations

First, this report was an observational, single-center and relatively small study. We did not examine whether the control of serum phosphate could slow CAC progression. Second, the follow-up times of these patients varied from 6 months to 87 months. Linear regression and geometric averages were used to evaluated CAC progression, which may have introduced errors.

## Abbreviations

MSCT: Multislice spiral computed tomography
CVD: Cardiovascular disease
PD: Peritoneal dialysis
ESRD: End-stage renal disease
CAC: Coronary artery calcification
CaCS: Coronary artery calcification score
iPTH: Intact parathyroid hormone
pro-BNP: Pro-B-type natriuretic peptide
hs-CRP: High-sensitivity C-reactive protein
Ccr: Creatinine clearance rate
nPCR: Normalized protein catabolic rate
BMI: Body mass index
BSA: Body surface area
HDL: High-density lipoprotein
LDL: Low-density lipoprotein
Lp (a): lipoprotein (a)
BUN: Blood urea nitrogen
CO_2_CP: Carbon dioxide combining power
Cp: The clearance of phosphate by the dialysate adjusted for the body surface area
Cca: The clearance of calcium by the dialysate adjusted for the body surface area
D4/P phosphate: The phosphate concentration in the dialysate at the 4^th^ hour / the plasma phosphate concentration
D/P phosphate: The mean phosphate concentration in the dialysate / the plasma phosphate concentration
D/P calcium: The mean calcium concentration in the dialysate / the plasma calcium concentration
